# Hospital market concentration and the use of mechanical circulatory support devices in acute myocardial infarction complicated by cardiogenic shock

**DOI:** 10.1186/s12913-021-07458-1

**Published:** 2022-01-19

**Authors:** Adam S. Vohra, Sun-Joo Jang, Dmitriy N. Feldman, Parag Goyal, Udhay Krishnan, Christopher Sciria, Jim W. Cheung, Luke K. Kim

**Affiliations:** 1 Department of Medicine, Division of Cardiology, Weill Cornell Cardiovascular Outcomes Research Group (CORG), 520 E 70th Street, Starr 4, New York, NY 10021 USA; 2 Yale New Haven Health, Bridgeport Hospital, Bridgeport, CT USA; 3 Division of Cardiology; Division of General Internal Medicine; Department of Medicine, Weill Cornell Medicine, New York, USA

**Keywords:** Hospital market concentration, Hospital consolidation, Cardiogenic shock, Acute myocardial infarction, Mechanical circulatory support

## Abstract

**Background:**

As health care markets in the United States have become increasingly consolidated, the role of market concentration on physician treatment behavior remains unclear. In cardiology, specifically, there has been evolving treatment of acute myocardial infarction complicated by cardiogenic shock (AMI-CS) with increasing use of mechanical circulatory support (MCS). However, there remains wide variation in it use. The role of market concentration in the utilization of MCS in AMI-CS is unknown. We examined the use of MCS in AMI-CS and its effect on outcomes between competitive and concentrated markets.

**Methods and results:**

We used the National Inpatient Sample to query patients admitted with AMI-CS between 2003 and 2009. The primary study outcome was the use of mechanical circulatory support. The primary study exposure was market concentration, measured using the Herfindahl-Hirschman Index, which was used to classify markets as unconcentrated (competitive), moderately concentrated, and highly concentrated. Baseline characteristics, procedures, and outcomes were compared for patients in differently concentrated markets. Multivariable logistic regression was used to examine the association between HHI and use of MCS.

**Results:**

There were 32,406 hospitalizations for patients admitted with AMI-CS. Patients in unconcentrated markets were more likely to receive MCS than in highly concentrated markets (unconcentrated 46.8% [5087/10,873], moderately concentrated 44.9% [2933/6526], and high concentrated 44.5% [6676/15,007], *p* < 0.01). Multivariable regression showed that patients in more concentrated markets had decreased use of MCS in patients in later years of the study period (2009, OR 0.64, 95% CI 0.44–0.94, *p* = 0.02), with no effect in earlier years. There was no significant difference in in-hospital mortality.

**Conclusion:**

Multivariable analysis did not show an association with market concentration and use of MCS in AMI-CS. However, subgroup analysis did show that competitive hospital markets were associated with more frequent use of MCS in AMI-CS as frequency of utilization increased over time. Further studies are needed to evaluate the effect of hospital market consolidation on the use of MCS and outcomes in AMI-CS.

**Supplementary Information:**

The online version contains supplementary material available at 10.1186/s12913-021-07458-1.

## Main text

### Background

During the last two decades, there has been a significant increase in the consolidation of hospitals and health systems due to health system mergers. Hospital consolidation reduces the number of hospitals in a market, increases concentration of hospital markets, and decreases market competition [1]. This has been driven largely by market and policy forces that have incentivized hospital consolidation to reduce costs by utilizing economies of scale and scope and improve outcomes by integrating care coordination [2, 3].

Previous studies have shown, however, that hospital consolidation may actually increase prices and health system costs [4, 5]. There is still a paucity of recent data on its effect on quality of care and outcomes, specifically in cardiovascular disease. While consolidation may theoretically lead to improvements of care through improved care coordination—including transfers to centers of excellence—these same forces could theoretically also reduce market incentives to improve care by disincentivizing hospitals from investing in innovations that provide a competitive edge [6]. For example, a recent study showed that robotic surgery is less commonly performed in concentrated markets, suggesting that in less concentrated, or more competitive markets, there may be more rapid deployment of new technologies [7].

In the field of cardiovascular disease, cardiogenic shock (CS) is a leading cause of mortality for patients with acute myocardial infarction (AMI) with an incidence of about 10% [8]. Despite advances in the treatment of AMI, in-hospital mortality for patients with CS remains high (~ 40–50%) [8–11]. Over the last 20 years, there has been an increased utilization of mechanical circulatory support devices (MCS) for CS [12, 13]. Given the lack of clear randomized data on the risk and benefits of MCS in CS, there is a lack of consensus with regards to the role of mechanical circulatory support, especially with intraaortic balloon pumps (IABP), in cardiogenic shock. Current American College of Cardiology / American Heart Association guidelines suggest that IABP can be useful in ST-elevation AMI patients who do not stabilize with pharmacologic therapy (Class IIb recommendation) [14], while European Society of Cardiology recommends against routine use (Class III recommendation) [15]. Because of this lack of clarity, there is wide variation in its use between hospitals, with previous studies showing that hospitals with higher utilization are more likely to be larger and less likely to be government-owned [16].

There are limited studies evaluating the effect of hospital market structure on utilization of MCS in AMI complicated by cardiogenic shock (AMI-CS) and resultant outcomes of these patients. We studied a time period with both increasing market concentration and evolving use of mechanical circulatory support with cardiogenic shock (2003–2009). We hypothesize that during this time period, more competitive (unconcentrated) markets will be associated with increased use of MCS devices for patients with AMI-CS. Using a nationally representative database, we examined the use of MCS devices in CS and its effect on in-hospital outcomes between concentrated and unconcentrated (or competitive) markets.

## Methods

### Data source

All data were obtained from the National Inpatient Sample (NIS), a publicly available, nationally representative database administered by the United States Agency for Healthcare Research and Quality (AHRQ) Healthcare Cost and Utilization Project (HCUP) [17]. Data was utilized from 2003 to 2009. The NIS is a 20% stratified sample of discharges from about 1000 hospitals. For the years utilized for this analysis, all discharges from sampled hospitals were included. Each hospitalization in the NIS includes all reported diagnosis and procedure codes based on the *International Classifications of Diseases, Ninth Revision, Clinical Modification* (*ICD-9-CM)*. Additional patient-level data included age, sex, and race. Hospital-level data included bed size, location (urban/rural), ownership, and teaching status. Prior to 2004, hospitals in a Metropolitan Statistical Area (MSA) were considered urban, while those outside an MSA were considered rural. Beginning with 2004, hospitals with a Core Based Statistical Area (CBSA) of Metropolitan or Division were considered urban, while those with a CBSA of Micropolitan or Rural were considered rural. Hospitals were considering teaching if they met one of the following criteria: 1) had a residency training approval by the Accreditation Council for Graduate Medical Education, 2) had membership in the Council of Teaching Hospitals, or 3) had a ratio of full-time equivalent interns and residents to beds of 0.25 or higher [17].

The Hospital Market Structure (HMS) file was linked to the NIS database for information on hospital market structure. The HMS data are updated every 3 years and last were updated in 2009 (state and hospital identifiers required for this supplemental file were removed from the NIS in 2012 to enhance confidentiality). Hospital market competition was assessed using the Herfindahl-Hirschman Index (HHI). The HHI is sum of squared market shares for all hospitals in the market. Market share is calculated by the number of discharges for a hospital divided by the total discharges from all hospitals in a market [18]. The market was defined using the variable radius market definition. For each hospital, the distance between the hospital and each patient’s ZIP code was calculated. ZIP codes were then ranked in ascending order according to distance and then were aggregated until 75% of the hospital’s discharges were attained. The distance between the hospital and the last ZIP code was used to determine the variable radius used to define that hospital’s market. Sensitivity analysis was conducted using variable radius of 90% of patient discharges to determine any effect of varying definitions of a hospital’s market [17]. Standard definitions of HHI determined by the Department of Justice and Federal Trade Commission to evaluate horizontal mergers and used in prior research were utilized: greater than 2500 was considered highly concentrated; 1500 to 2500 was considered moderately concentrated; and less than 1500 was considered unconcentrated or competitive [7, 19]. (See Figure S1)

Institutional review board approval and informed consent was not required since data was derived from a deidentified administrative database.

### Study population

From 2003 to 2009 all hospitalizations for patients with the diagnosis of cardiogenic shock (*ICD-9-CM* 785.31) and AMI (*ICD-9-CM* 410.11–410.91) in any position were queried from the NIS database [20]. Hospitals performing less than 10 percutaneous coronary interventions (PCI) per year were excluded from analysis in order to select for hospitals with cardiac catheterization laboratories. Hospitals unable to perform PCI were assumed not to have the capacity to place MCS devices. Patients were classified based on demographics such as age, sex, and race. Comorbidities were identified using 29 Elixhauser clinical and procedural variables defined by the admitting ICD-9 codes. Administrative codes were used to determine if patients underwent coronary angiography, right heart catheterization (RHC), PCI, or coronary artery bypass grafting (CABG) during index hospitalization (see Table S1).

### Study exposures and outcomes

The primary exposure of interest was HHI (as described above). For our primary analysis, variable radius of 75% of hospital discharges were used. Sensitivity analysis was performed with variable radius of 90%. Patient-level covariates included age, sex, race, comorbidities, and receipt of procedures (coronary angiography, PCI, or CABG) as noted above. Hospital-level covariates included bed size, teaching status, location, and hospital control.

The primary outcome variable was the use of MCS. MCS devices included percutaneous devices (*ICD-9-CM* 37.68), non-percutaneous devices (*ICD-9-CM* 37.60 and 37.65), intra-aortic balloon pumps (IABP) (*ICD-9-CM* 37.61), and extracorporeal membrane oxygenation (ECMO) (*ICD-9-CM* 39.65 and 39.66). Percutaneous devices include devices such as Impella (Abiomed Inc., Danvers, MA) and TandemHeart (CardiacAssist, Pittsburgh, PA), while nonpercutaneous devices include Thoratec paracorporeal ventricular assist devices (Thoratec Inc., Pleasanton, CA), AB5000 (Abiomed, Inc., Danvers, MA), BVS5000 (Abiomed, Inc., Danvers, MA), and Centrimag (Thoratec Inc., Pleasanton, CA) [12]. The secondary outcome variable was in-hospital mortality which was captured through the NIS database.

### Statistical analysis

Patient-level characteristics such as age, sex, race, and comorbidities were compared across levels of HHI with 1-way ANOVA for continuous variables and chi-square tests for categorical variables. Admission data, including rates of coronary angiography, RHC, PCI, and CABG as well hospital characteristics were also compared across levels of HHI using chi-square tests. Receipt of MCS and in-hospital mortality were compared across levels of HHI using chi-square tests. A multivariable logistic mixed effect model was used to model the receipt of MCS according to patient, admission, and hospital characteristics, including HHI. Hospital was included as a random effect to account for clustering of hospitalizations by hospital. Given change in treatment approaches for CS during the study time period, analysis was re-conducted by year (2003, 2006, and 2009 chosen to align with HMS updates) to evaluate temporal trends. All analyses were performed with R version 3.6.3.

## Results

### Patient and hospital characteristics

This study included a total of 32,406 hospitalizations at 892 hospitals between 2003 and 2009 for patients with AMI complicated by cardiogenic shock. Of the hospitalizations analyzed, 10,873 (33.6%) were in hospitals in unconcentrated (competitive) markets, 6526 (20.1%) in hospitals in moderately concentrated markets, and 15,007 (46.3%) in hospitals with highly concentrated markets. Of the hospitals included, initially 247 (27.7%) were in unconcentrated (competitive) markets, 172 (19.3%) in moderately concentrated markets, and 473 (53.0%) in highly concentrated markets. Thirty-six hospitals had a change in market concentration category during the study period. Patients in differently concentrated markets were compared by age and comorbid conditions (see Table 1). Patients in unconcentrated markets were more likely to have congestive heart failure, valvular heart disease, pulmonary circulation disorders, and cancer. Patients in moderately concentrated or highly concentrated markets were more likely to have chronic obstructive pulmonary disease, obesity, and hypertension. Teaching status was more common in competitive markets (74.6%) than in moderately concentrated (57.8%) or highly concentrated (36.6%) markets. The majority of hospitals had large bed-size and urban location across all market concentrations (Table 2).

**Table 1:** Baseline Patient Characteristics (2003–2009)

	Competitive / Unconcentrated	Moderately Concentrated	Highly Concentrated	***p***-value
	(***n*** = 10,873)	(***n*** = 6526)	(***n*** = 15,007)	
**Age (years)**	68.2	68.7	69.5	< 0.01
**Median household income**				< 0.01
First quartile	2657 (24.4%)	1529 (23.4%)	4094 (27.3%)	
Second quartile	2500 (23.0%)	1318 (20.2%)	4226 (28.2%)	
Third quartile	2705 (24.9%)	1593 (24.4%)	3793 (25.3%)	
Fourth quartile	2729 (25.1%)	1924 (29.5%)	2504 (16.7%)	
Unknown	282 (2.6%)	162 (2.5%)	390 (2.6%)	
**Payer status**				< 0.01
Medicare	6286 (57.8%)	3890 (59.6%)	9534 (63.5%)	
Medicaid	763 (7.0%)	382 (5.9%)	726 (4.8%)	
Private insurance	2838 (26.1%)	1768 (27.1%)	3542 (23.6%)	
Self-pay / no charge / other	986 (9.1%)	486 (7.4%)	1205 (8.0%)	
**Comorbid conditions**				
Congestive heart failure	6000 (55.2%)	3477 (53.3%)	8091 (53.9%)	0.03
Valvular disease	2142 (19.7%)	1143 (17.5%)	2879 (19.2%)	< 0.01
Pulmonary circulation disorders	564 (5.2%)	280 (4.3%)	647 (4.3%)	< 0.01
Peripheral vascular disease	1132 (10.4%)	628 (9.6%)	1439 (9.6%)	0.06
Paralysis	108 (1.0%)	52 (0.8%)	131 (0.9%)	0.37
Neurologic disease	599 (5.5%)	343 (5.3%)	826 (5.5%)	0.73
Chronic obstructive pulmonary disease	2162 (19.9%)	1464 (22.4%)	3641 (24.3%)	< 0.01
Diabetes mellitus, uncomplicated	2284 (21.0%)	1408 (21.6%)	3115 (20.8%)	0.4
Diabetes mellitus, with chronic complications	559 (5.1%)	331 (5.1%)	692 (4.6%)	0.11
Hypothyroidism	532 (4.9%)	342 (5.2%)	814 (5.4%)	0.16
Chronic kidney disease	1821 (16.7%)	1179 (18.1%)	2543 (16.9%)	0.06
Liver disease	142 (1.3%)	85 (1.3%)	189 (1.3%)	0.94
Lymphoma	62 (0.6%)	33 (0.5%)	92 (0.6%)	0.63
Solid tumor without metastasis	304 (2.8%)	142 (2.2%)	371 (2.5%)	0.03
Metastatic cancer	128 (1.2%)	62 (1.0%)	165 (1.1%)	0.38
Collagen vascular disease	145 (1.3%)	98 (1.5%)	228 (1.5%)	0.44
Coagulopathy	1337 (12.3%)	671 (10.3%)	1336 (8.9%)	< 0.01
Obesity	508 (4.7%)	365 (5.6%)	828 (5.5%)	< 0.01
Weight loss	641 (5.9%)	423 (6.5%)	804 (5.4%)	< 0.01
Fluid and electrolyte disorders	3973 (36.5%)	2435 (37.3%)	5278 (35.2%)	< 0.01
Chronic blood loss anemia	188 (1.7%)	118 (1.8%)	279 (1.9%)	0.74
Deficiency anemia	1325 (12.2%)	922 (14.1%)	1753 (11.7%)	< 0.01
Alcohol abuse	347 (3.2%)	168 (2.6%)	452 (3.0%)	0.07
Drug abuse	168 (1.5%)	85 (1.3%)	172 (1.1%)	0.02
Psychoses	176 (1.6%)	93 (1.4%)	189 (1.3%)	0.05
Depression	294 (2.7%)	158 (2.4%)	380 (2.5%)	0.49
Hypertension	4746 (43.6%)	3003 (46.0%)	6702 (44.7%)	0.01

**Table 2:** Baseline Hospital Characteristics (2003–2009)

	Competitive / Unconcentrated	Moderately Concentrated	Highly Concentrated	***p***-value
	(***n*** = 10,873)	(***n*** = 6526)	(***n*** = 15,007)	
**Hospital bed size**				< 0.01
Small	793 (7.3%)	532 (8.2%)	730 (4.9%)	
Medium	2521 (23.2%)	1028 (15.8%)	3069 (20.5%)	
Large	7529 (69.2%)	4959 (76.0%)	11,135 (74.2%)	
Unknown	30 (0.3%)	7 (0.1%)	73 (0.5%)	
**Teaching status**	8112 (74.6%)	3771 (57.8%)	5497 (36.6%)	< 0.01
**Location**				< 0.01
Urban	10,591 (97.4%)	6510 (99.8%)	13,743 (91.6%)	
Rural	252 (2.3%)	9 (0.1%)	1191 (7.9%)	
Unknown	30 (0.3%)	7 (0.1%)	73 (0.5%)	
**Hospital control**				< 0.01
Government or private	8676 (79.8%)	4164 (63.8%)	7544 (50.3%)	
Government, nonfederal	40 (0.4%)	50 (0.8%)	1122 (7.5%)	
Private, non-profit	1410 (13.0%)	1411 (21.6%)	3811 (25.4%)	
Private, investor-own	717 (6.6%)	885 (13.6%)	1816 (12.1%)	
Private, other	0 (0.0%)	9 (0.1%)	641 (4.3%)	
Unknown	30 (0.3%)	7 (0.1%)	73 (0.5%)	

### Patient-level analysis

Patients treated at hospitals in unconcentrated markets were more likely to get MCS than those in concentrated markets (unconcentrated 46.8% [5087/10,873], moderately concentrated 44.9% [2933/6526], and high concentrated 44.5% [6676/15,007], *p* < 0.01). In all markets, the predominant form of MCS was IABP encompassing 99% of all MCS devices. While there were slightly higher rates of coronary angiography and PCI in highly concentrated markets, there were higher rates of RHC, CABG, and ECMO in unconcentrated markets. There was no significant difference in in-hospital mortality between differently concentrated markets (Table 3).

**Table 3:** Rate of Procedures, Mechanical Circulatory Support, and Mortality (2003–2009)

	Competitive / Unconcentrated	Moderately Concentrated	Highly Concentrated	***p***-value
	(***n*** = 10,873)	(***n*** = 6526)	(***n*** = 15,007)	
**Mechanical circulatory support**	5087 (46.8%)	2933 (44.9%)	6676 (44.5%)	< 0.01
Percutaneous devices	45 (0.4%)	15 (0.2%)	7 (0.1%)	< 0.01
Nonpercutaneous devices	42 (0.4%)	21 (0.3%)	26 (0.2%)	< 0.01
Intraaortic balloon pump	5024 (46.2%)	2918 (44.7%)	6647 (44.3%)	< 0.01
Extracorporeal membrane oxygenation	68 (0.6%)	4 (0.1%)	13 (0.1%)	< 0.01
**Procedures**				
Coronary angiography	7103 (65.3%)	4280 (65.6%)	10,037 (66.9%)	0.02
Right heart catheterization	441 (4.1%)	144 (2.2%)	238 (1.6%)	< 0.01
Percutaneous coronary intervention	4753 (43.7%)	2719 (41.7%)	6748 (45.0%)	< 0.01
Coronary artery bypass graft	2251 (20.7%)	1389 (21.3%)	2489 (16.6%)	< 0.01
In-hospital mortality	3997 (36.8%)	2434 (37.3%)	5545 (36.9%)	0.77

In a multivariable logistic mixed effect model using all years (2003–2009), patients with congestive heart failure, valvular disease, diabetes mellitus, coagulopathy, and fluid and electrolytes disturbances (see Table 4), were more likely to receive MCS. After controlling for hospital level clustering with a mixed effect model with hospital as a random intercept, hospital bed size, control, teaching status, or location had no additional effect on receipt of MCS. Patients who underwent coronary angiography, PCI, or CABG during hospitalization were more likely to receive MCS (see Table 4).

**Table 4:** Multivariable Logistic Mixed Effects Model, Patient Characteristics (2003–2009)

Variable	Odds Ratio	Confidence interval	***p***-value
**PRIMARY EXPOSURE VARIABLE**			
**Herfindal-Hirschman Index (variable radius 75%)**	0.91	0.76–1.10	0.34
**COVARIATES OF INTEREST**			
**Age**	0.98	0.98–0.99	< 0.01
**Female**	0.76	0.72–0.80	< 0.01
**Race**			
*White (reference)*	REF	REF	REF
*Black*	0.79	0.69–0.89	< 0.01
*Hispanic*	0.98	0.87–1.10	0.77
*Asian or Pacific Islander*	1.18	0.98–1.41	0.08
*Native American*	1.04	0.71–1.51	0.85
*Other / Unknown*	1.06	0.98–1.15	0.14
**Hospital Bedsize**			
*Small (reference)*	REF	REF	REF
*Medium*	0.98	0.82–1.16	0.79
*Large*	1.04	0.89–1.23	0.61
**Hospital Control**			
*Government or private, collapsed (reference)*	REF	REF	REF
*Government, nonfederal, public*	0.88	0.68–1.13	0.31
*Private, non-profit, voluntary*	0.87	0.74–1.02	0.08
*Private, invest-own*	0.99	0.83–1.17	0.87
*Private, collapsed*	0.96	0.66–1.39	0.83
**Teaching Hospital**	0.93	0.81–1.06	0.28
**Urban**	0.96	0.74–1.24	0.77
**Procedures**			
*Left heart catherization*	5.07	4.74–5.43	< 0.01
*Percutaneous coronary intervention*	2.62	2.46–2.79	< 0.01
*Coronary artery bypass graft*	4.34	4.03–4.68	< 0.01
**Comorbid Conditions**			
*Congestive heart failure*	1.25	1.19–1.33	< 0.01
*Valvular disease*	1.14	1.07–1.22	< 0.01
*Peripheral vascular disease*	0.70	0.64–0.76	< 0.01
*Neurologic disease*	0.74	0.65–0.83	< 0.01
*Chronic obstructive pulmonary disease*	0.76	0.71–0.81	< 0.01
*Diabetes mellitus, uncomplicated*	1.09	1.02–1.17	< 0.01
*Chronic kidney disease*	0.83	0.77–0.90	< 0.01
*Liver disease*	0.76	0.59–0.98	0.03
*Coagulopathy*	1.44	1.31–1.57	< 0.01
*Fluid and electrolyte disorders*	1.09	1.03–1.15	< 0.01
*Hypertension*	0.88	0.83–0.93	< 0.01

### Market concentration

**Fig. 1:**
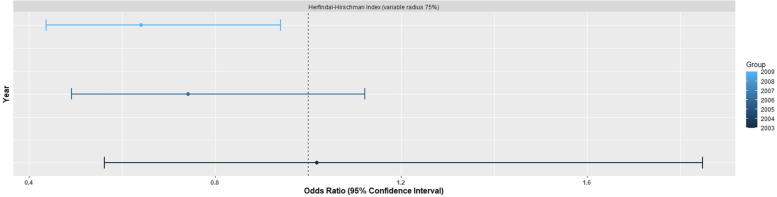
Odds Ratio of Herfindal-Hirschman Index (variable radius 75%) by Year. Multivariable mixed effect model for patients with acute myocardial infarction complicated by cardiogenic shock showed that market concentration was a significant predictor of receiving mechanical circulatory support for patients in 2009. Market concentration was not significant for patients in 2003 or 2006

In multivariable logistic mixed effect model using all years (2003–2009), market concentration did not show any statistically significant effect on the use of MCS (See Tables S2 and S3 for full regression models). Multivariable logistic mixed effect model was re-analyzed separately by year to assess temporal trends. Market concentration did not show any effect on the use of MCS during analysis of patients from 2003 or 2006. However, for 2009, the mixed effect model showed that patients within markets with higher HHI (more concentrated markets) were less likely to receive MCS than those in markets with lower HHI (more unconcentrated / competitive ones) (OR 0.64, 95% CI 0.44–0.94, *p* = 0.02, see Fig. 1). Remaining predictors showed no additional differences across years. There was a substantial increase in the volume of MCS across the study period, especially percutaneous devices such as Impella (Abiomed Inc., Danvers, MA) (see Fig. S2). Sensitivity analysis was performed using multivariable logistic mixed effect models for HHI variable radius 90%, but did not significantly change our results.

## Discussion

Our study demonstrated, that in addition to patient and hospital characteristics, regional market forces are associated with treatment decisions for patients admitted with AMI-CS. We showed that during our study period (2003–2009), in unadjusted analysis, patients in unconcentrated (or competitive) markets were more likely to receive MCS when admitted with AMI-CS than those in concentrated markets. In mixed-effect multivariable regression analysis, adjusting for patient and hospital factors, our study found that in more contemporary years (2009), market concentration was a significant factor in receipt of MCS.

Prior research has suggested that hospital market forces are associated with treatment decisions, especially with regard to diffusion of new technologies [7, 21, 22]. Studies have shown that in unconcentrated markets, patients are more likely to undergo laparoscopic colectomy (compared to open) for colon cancer [21], more likely to undergo endovascular repair (compared to open) for abdominal aortic aneurysm [22], and more likely to undergo robotic-assisted surgery for genitourinary and gynecologic surgery [7]. Notably, these studies controlled for both patient and hospital characteristics, suggesting a true effect from market concentration. Wright et al. showed that patients in unconcentrated markets were more likely to undergo robotic-assisted surgery. However, once analysis was restricted to hospitals that already performed robotic-assisted surgery, there was no association. This suggests that while market forces may influence decision to acquire technology—such as a surgical robot—it may not affect patient care decisions once the technology is acquired.

AMI-CS is an emergent condition requiring timely management. We hypothesized that hospitals in unconcentrated markets will have increased investment in MCS and be associated with higher utilization of MCS. Our study was conducted using data during a time of evolving management of AMI-CS with increasing use of percutaneous MCS and showed that overall, hospital market concentration did not have any effect on the receipt of MCS in the earlier years of the study period. However, in the later years of the study, while we saw an increase in utilization of MCS among all hospitals, patients in unconcentrated markets became more likely to receive MCS compared to those in concentrated ones. This effect was seen regardless of other hospital characteristics such as location, bed-size, and teaching status. Abiomed obtained 510(k) clearance from FDA for the Impella percutaneous device (Abiomed Inc., Danvers, MA) in May 2008. NIS data shows a doubling of use of percutaneous devices in patients with AMI-CS between 2008 and 2009 which may be driving early adoption in more competitive market (See Fig. S2).

Health care policy over the past two decades encourages consolidation of markets—leading to increased market concentration—with the formation of accountable care organizations and use of bundled payments [4]. While the goal of these policies has been improvement in coordination of care, there are likely unintended effects on costs of care and patient outcomes [23]. One of these effects may be a decreased diffusion of new technologies, both proven (but underutilized) and unproven (and yet to demonstrate benefit). Expanded uses of proven technologies may improve patient outcomes, while increased use of unproven technologies may increase healthcare costs, without an effect on patient outcomes [24]. Our study is the first, that we are aware, to evaluate these effects of hospital market concentration on use of cardiovascular technology. These findings may be useful in informing health care policy and federal antitrust efforts to mitigate negative effects of hospital consolidation.

There are a number of strengths to this study. First, the NIS is a nationwide database that is representative of the United States population. We were able to capture patients with cardiogenic shock in a crucial time period during national trends in increased market concentration and increased use of mechanical circulatory support. However, our study also has a number of limitations. First, while we were able to capture a unique period in the evolution of mechanical circulatory support, analysis of more contemporary data outside the NIS (which does not provide hospital market level data after 2009) is needed to analyze more recent effects. These recent effects will be better suited to evaluate the effect of market concentration once technologies (such as Impella) are more broadly used. Second, we used an administrative database and were unable to account for potential unmeasured confounders, including severity of presenting illness, medications, and socioeconomic factors. It is possible that there may be bias toward sicker patients in competitive markets that may not be realized in administrative data and might confound the effects of market concentration. Third, the NIS is limited to a sampling of only 20% of hospitals in the United States. Random sampling may have affected our results if hospitals selected in a particular market were biased toward more or less frequent MCS use compared to other representative hospitals in that market. Our study, however, captured over 800 hospitals and controlled for hospital factor such as bed size, teaching status, and location while likely minimized any effect of random sampling. Fourth, there is variation in the literature in identifying patients with AMI-CS from administrative data. We used a method from prior literature [16, 20], that uses encounters that have *ICD-9-CM* codes for cardiogenic shock or myocardial infarction in any position in the NIS databases. This method increases sensitivity for capturing cases of AMI-CS, but may capture cases of cardiogenic shock that are not due to myocardial infarction. Other literature, limits encounters to those that have myocardial infarction as the primary diagnosis [8, 25]. Finally, HHI was used as an indicator for market concentration. While HHI is a validated metric to evaluate market concentration, it may not fully capture other important market factors, including hospital affiliations.

## Conclusions

Multivariable analysis did not show an association with market concentration and use of MCS in AMI-CS in our study time period (2003–2009). However, subgroup analysis by year did show that competitive hospital markets were associated with more frequent use of MCS in AMI-CS as frequency of utilization increased over time. Further studies are needed to evaluate the effect of hospital market consolidation on the use of MCS and outcomes in AMI-CS.

## Supplementary Information

**Additional file 1..**

## Data Availability

The datasets analyzed during the current study are available through the Healthcare Cost and Utilization Project (https://www.hcup-us.ahrq.gov/) [17].
