# Supplementary material for: Hospital market concentration and the use of mechanical circulatory support devices in acute myocardial infarction complicated by cardiogenic shock
Source: BMC Health Serv Res. 2022 Jan 19;22:89. doi: 10.1186/s12913-021-07458-1 (PMC8772168; doi:10.1186/s12913-021-07458-1)

SUPPLEMENTAL MATERIAL

"Hospital Market Concentration and the Use of Mechanical Circulatory Support Devices in Acute Myocardial Infarction Complicated by Cardiogenic Shock"

Figure S1: Herfindahl-Hirschman Index. (A) Definition of HHI. HHI is defined as the square of sum of market shares of all hospitals in a market. **(B) Defining Hospital Radius.** A hospital's market can be defined as either a fixed or variable radius. Fixed radius evaluates all hospitals within a certain distance of the hospital. Variable radius evaluates a distance that encompasses a percentage of a hospital's discharges.

A

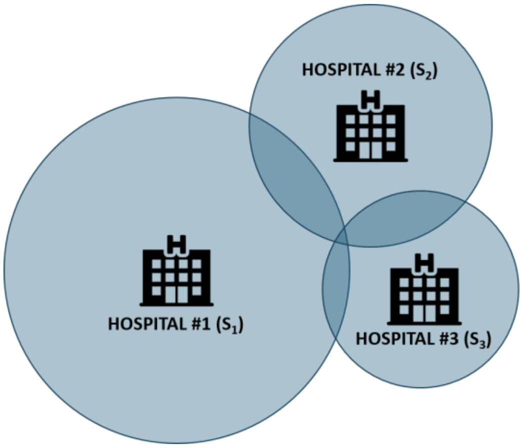

$$HHI = \sum_{i=1}^{N} S_i^2$$

$HHI = Herfindahl - Hirschman\ Index$

$S_i = market\ share\ of\ firm\ i$

B

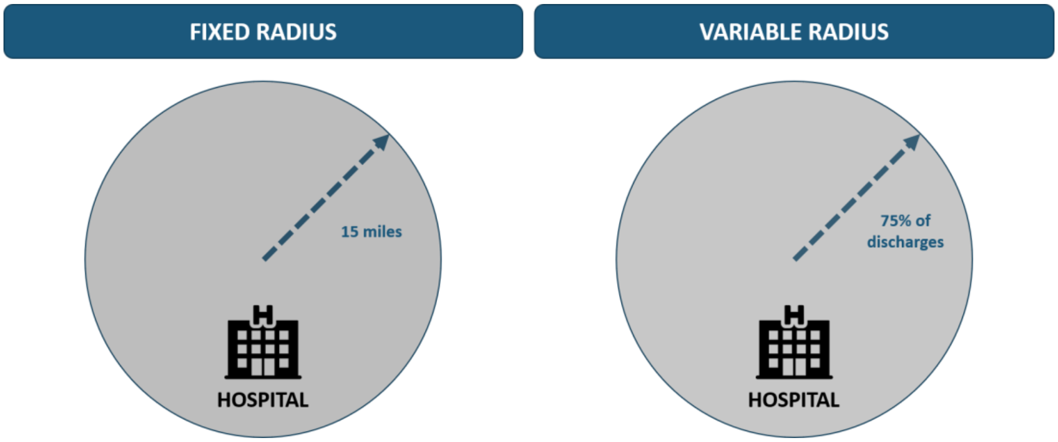

Table S1: Administrative Codes Used to Define Variables

Variable Name	ICD-9-CM Code
**Percutaneous devices**	37.68
**Non-percutaneous devices**	37.60, 36.65
**IABP**	37.61
**ECMO**	39.65, 39.66
**Coronary angiography**	37.22, 37.23, 88.52, 88.53, 88.54, 88.55, 88.56, 88.57
**PCI**	00.66, 17.55, 36.01, 36.02, 36.05, 36.06, 36.07
**CABG**	36.10-36.17, 36.19
**Right heart catheterization**	37.21
**Congestive heart failure**	398.91, 402.01, 402.11, 402.91, 404.01, 404.03, 404.11, 404.13, 404.91, 404.93, 428.0- 428.9
**Valvular disease**	093.20- 093.24, 394.0- 397.1, 397.9, 424.0- 424.99, 746.3- 746.6, V42.2, V43.3
**Pulmonary circulation disorders**	415.0, 415.11- 415.19, 416.0- 416.9, 417.0-417.9
**Peripheral vascular disorders**	440-440.9, 441.00- 441.9, 442.0- 442.9, 443.1- 443.9, 444.21- 444.22, 447.1,449, 557.1, 557.9, V43.4
**Paralysis**	342.0- 344.9
**Other neurological disorders**	330.0- 331.9, 332.0, 333.4, 333.5, 333.71, 333.72, 333.79, 333.85, 333.94, 334.0- 335.9, 338.0, 340, 341.1- 341.9, 345.00- 345.11, 345.2- 345.3, 345.40- 345.91, 347.00- 347.01, 347.10- 347.11, 649.40- 649.44, 768.7, 768.70, 768.71, 768.72, 768.73, 780.3, 780.31, 780.32, 780.33, 780.39, 780.97, 784.3
**Chronic pulmonary disease**	490-492.8, 493.00- 493.92, 494-494.1, 495.0-505, 506.4
**Diabetes, uncomplicated**	249.00- 249.31, 250.00- 250.33, 648.00- 648.04
**Diabetes with chronic complications**	249.40- 249.91, 250.40- 250.93, 775.1
**Hypothyroidism**	243-244.2, 244.8, 244.9
**Renal failure**	403.01, 403.11, 403.90, 403.91, 404.02, 404.03, 404.12, 404.13, 404.92, 404.93, 585.3, 585.4, 585.5, 585.6, 585.9, 586, V42.0, V45.1, V45.11, V45.12, V56.0- V56.32, V56.8
**Liver disease**	070.22, 070.23, 070.32, 070.33 , 070.44, 070.54, 456.0, 456.1, 456.20, 456.21, 571.0, 571.2, 571.3, 571.40- 571.49, 571.5, 571.6, 571.8, 571.9, 572.3, 572.8, 573.5, V42.7
**Peptic ulcer disease excluding bleeding**	531.41, 531.51, 531.61, 531.70, 531.71, 531.91, 532.41, 532.51, 532.61, 532.70, 532.71, 532.91, 533.41, 533.51, 533.61, 533.70, 533.71, 533.91, 534.41, 534.51, 534.61, 534.70, 534.71, 534.91
**Acquired immune deficiency syndrome**	042.0
**Lymphoma**	200.00- 202.38, 202.50- 203.01, 203.02- 203.82, 203.8- 203.81, 238.6, 273.3
**Metastatic cancer**	196.0- 199.2, 209.70, 209.71, 209.72, 209.73, 209.74, 209.75, 209.79, 789.51
**Solid tumor without metastasis**	140.0- 172.9, 174.0- 175.9, 179-195.8, 209.00- 209.24, 209.25- 209.3, 209.30- 209.36, 258.01- 258.03
**Rheumatoid arthritis/collagen vascular diseases**	701.0, 710.0- 710.9, 714.0- 714.9, 720.0- 720.9, 725
**Coagulopathy**	286.0- 286.9, 287.1, 287.3- 287.5, 289.84, 649.30- 649.34
**Obesity**	278.0, 278.00, 278.01, 278.03, 649.10- 649.14, 793.91, V85.30- V85.39, V85.41- V85.45, V85.54
**Weight loss**	260-263.9, 783.21, 783.22
**Fluid and electrolyte disorders**	276.0- 276.9
**Chronic blood loss anemia**	280.0, 648.20- 648.24
**Deficiency anemias**	280.1- 281.9, 285.21- 285.29, 285.9
**Alcohol abuse**	291.0- 291.3, 291.5, 291.8, 291.81, 291.82, 291.89, 291.9, 303.00- 303.93, 305.00- 305.03
**Drug abuse**	292.0, 292.82- 292.89, 292.9, 304.00- 304.93, 305.20- 305.93, 648.30- 648.34
**Psychoses**	295.00- 298.9, 299.10, 299.11
**Depression**	300.4, 301.12, 309.0, 309.1, 311
**Hypertension, complicated and uncomplicated**	401.1, 401.9, 642.00- 642.04

Table S2: Multivariable Logistic Mixed Effects Model, Patient Characteristics (2003 – 2009)

Variable	Odds Ratio	Confidence interval	p-value
**Age**	0.98	0.98 – 0.99	<0.01
**Female**	0.76	0.72 – 0.80	<0.01
**Race**			
White (reference)	REF	REF	REF
Black	0.79	0.69 – 0.89	<0.01
Hispanic	0.98	0.87 – 1.10	0.77
Asian or Pacific Islander	1.18	0.98 – 1.41	0.08
Native American	1.04	0.71 – 1.51	0.85
Other / Unknown	1.06	0.98 – 1.15	0.14
**Comorbid Conditions**			
Congestive heart failure	1.25	1.19 – 1.33	<0.01
Valvular disease	1.14	1.07 – 1.22	<0.01
Pulmonary circulation disorders	1.0	0.88 – 1.13	0.99
Peripheral vascular disease	0.70	0.64 – 0.76	<0.01
Paralysis	0.84	0.63 – 1.12	0.24
Neurologic disease	0.74	0.65 – 0.83	<0.01
Chronic obstructive pulmonary disease	0.76	0.71 – 0.81	<0.01
Diabetes mellitus, uncomplicated	1.09	1.02 – 1.17	<0.01
Diabetes mellitus, with chronic complications	0.94	0.82 – 1.06	0.30
Hypothyroidism	0.98	0.86 – 1.10	0.69
Chronic kidney disease	0.83	0.77 – 0.90	<0.01
Liver disease	0.76	0.59 – 0.98	0.03
Peptic ulcer disease	0.00	0.00 - NA	0.94
Acquired immune deficiency syndrome	0.76	0.38 – 1.53	0.44
Lymphoma	0.65	0.45 – 0.94	0.02
Solid tumor without metastasis	0.92	0.76 – 1.12	0.41
Metastatic cancer	0.74	0.54 – 1.00	0.05
\Collagen vascular disease	0.81	0.65 – 1.01	0.07
Coagulopathy	1.44	1.31 – 1.57	<0.01
Obesity	1.05	0.93 – 1.18	0.44
Weight loss	0.97	0.86 – 1.10	0.65
Fluid and electrolyte disorders	1.09	1.03 – 1.15	<0.01
Chronic blood loss anemia	0.92	0.75 – 1.12	0.42
Deficiency anemia	0.95	0.88 – 1.03	0.24
Alcohol abuse	0.86	0.74 – 1.00	0.05
Drug abuse	0.83	0.66 – 1.05	0.12
Psychoses	0.94	0.74 – 1.16	0.50
Depression	0.84	0.71 – 0.99	0.04
Hypertension	0.88	0.83 – 0.93	<0.01

Table S3: Multivariable Logistic Mixed Effects Model, Admission and Hospital Characteristics (2003 – 2009)

Variable	Odds Ratio	Confidence interval	p-value
**Admission Type**			
Emergency (reference)	REF	REF	REF
Urgent	1.01	0.94 – 1.09	0.78
Elective	1.19	1.07 – 1.32	<0.01
Trauma Center	0.81	0.41 – 1.60	0.55
Other / Unknown	1.06	0.92 – 1.22	0.45
**Weekend Admission**	0.98	0.92 – 1.04	0.49
**Hospital Bedsize**			
Small (reference)	REF	REF	REF
Medium	0.98	0.82 – 1.16	0.79
Large	1.04	0.89 – 1.23	0.61
**Hospital Control**			
Government or private, collapsed (reference)	REF	REF	REF
Government, nonfederal, public	0.88	0.68 – 1.13	0.31
Private, non-profit, voluntary	0.87	0.74 – 1.02	0.08
Private, invest-own	0.99	0.83 – 1.17	0.87
Private, collapsed	0.96	0.66 – 1.39	0.83
**Teaching Hospital**	0.93	0.81 – 1.06	0.28
**Urban**	0.96	0.74 – 1.24	0.77
**Procedures**			
Left heart catherization	5.07	4.74 – 5.43	<0.01
Percutaneous coronary intervention	2.62	2.46 – 2.79	<0.01
Coronary artery bypass graft	4.34	4.03 – 4.68	<0.01
**Herfindal-Hirschman Index (variable radius 75%)**	0.91	0.76 – 1.10	0.34

Figure S2: Mechanical Circulatory Support by Year (2003 – 2009). Mechanical circulatory support utilization has increased over the study time period including substantial increase in percutaneous devices and extracorporeal membrane oxygenations. The rise in percutaneous devices, such as Impella, has been most prominent in competitive markets.

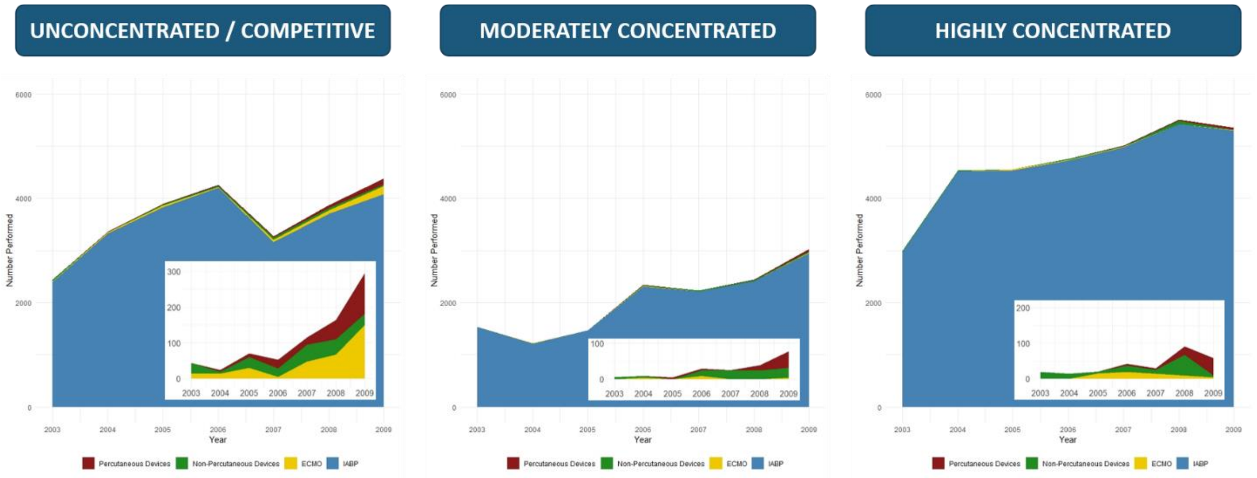

Supplement: Supplementary file 1 — Additional file 1.. [file 12913_2021_7458_MOESM1_ESM.pdf]
